# The Spread of Antisepsis

**Published:** 1888-10

**Authors:** 


### The Spread of Antisepsis.

The advocates of antisepsis have good cause for hearty self and mutual congratulation, in looking over the vast results achieved in the last few years. Time was, and not so very long ago, when this great reform was wholly confined to its inventor and his immediate friends and adherents. From this numerically small beginning it has spread, by sheer force of merit, until there is not a civilized country which has not felt its beneficial influence. Against it have been arrayed sectional hate and professional prejudice. Against it stood every man who was wedded to any other system of treatment whatever—every man who was unwilling to acknowledge that his past surgical life had to a certain extent, been a mistake. Not the least of its opponents has been that compound of dislike and novelty, respect for old traditions, intense prejudice and laziness, which is called " old fogyism."

Against these and other obstacles antisepsis has made its way —not by compulsion, but appealing rather to the conscience of the profession ; only needing a fair trial to be appreciated ; never losing a true convert, until the completeness of its conquest is astonishing even to enthusiasts.

The advantage of such a uniform practice as this, both to profession and people, is incalculable. To the former it is a surgical volapuk—a common standard by which one's own work and that of his brethren can be estimated and compared ; to the latter it offers a reasonable assurance that they will receive the best of treatment, no matter into whose hands they may fall.

By no means the least gratifying feature of this reform is the stand taken by our brethren of the country and smaller towns. It was to be expected that hospital surgeons everywhere, and practitioners in larger cities, where the results of antisepsis were forced upon their attention, would fall promptly in to line if only as a matter of self protection. But the fact that men who are masters of the situation in their own localities, should voluntarily array themselves on the right side, is only another proof of their professional intelligence and conscientiousness.

It has lately been the duty of the writer to take surgical cognizance of some twenty cases, injured in a railroad accident. They were treated by half a dozen physicians from three different towns. In not one of these cases was there any suppuration, nor any other ill result attributable to treatment.

